# Testing the impact of recruitment message content on open rate and consent rate for population-based genomic screening

**DOI:** 10.1017/cts.2025.10147

**Published:** 2025-09-15

**Authors:** Carolina Liskey, Daniel Judge, Kelly J. Hunt, Samantha Norman, Julia Wakser, John Clark, Wei Ding, Leslie A. Lenert, Caitlin G. Allen

**Affiliations:** 1 Medical University of South Carolina, Charleston, SC, USA; 2 Wake Forest University School of Medicine, Department of Implementation Science, Winston-Salem, NC, USA

**Keywords:** Genomics, genetic testing, recruitment methods, digitals tools, inclusivity of participation

## Abstract

**Background:**

Digital tools offer promising solutions to improve eligibility screening, recruitment, and retention in research, particularly in human genetic studies where representative sampling is critical. SMS text messaging has been found effective in population-based survey research, but evidence of its impact on genetic study recruitment – and how it varies by demographics – is limited.

**Objective:**

We examined the effect of tailored SMS messages on enrollment in a population-based genomic screening study. We assessed differences in message open and consent rates across four message types and explored how these outcomes varied by demographic factors.

**Methods:**

Participants were randomized to receive one of four SMS messages emphasizing different social values: generic, individual impact, community impact, or research discoveries. We calculated descriptive statistics for open and consent rates and used generalized linear logistic regression and Pearson’s Chi-Square Test to assess demographic differences.

**Results:**

Among 15,977 messages sent, 2.4% were opened (*n* = 382), and 35.3% of those who opened consented (*n* = 135). Females were more likely than males to open (3.1% vs. 1.6%) and consent (1.1% vs. 0.5%). Individuals aged 30–39 had the highest open rate (3.4%), and those 40–49 had the highest consent rate (1.6%). Message type was not significantly associated with open or consent rates.

**Conclusion:**

Sociodemographic factors were more predictive of engagement than message content. Tailoring messages by demographic group may improve recruitment in genomic studies. Future research should explore the drivers of participant engagement in digital recruitment strategies.

## Background

Recruitment and participation have been a continuous challenge for clinical and translational trials [1,2]. Digital tools promise to optimize and improve the eligibility, recruitment, and retention process in research [3,6]. Use of digital tools in research has grown in both volume and type [6]. This promise of digital tools is based on the successful use of digital tools in non-medical areas, such as recruiting volunteers for grassroots political organizations [7]. The effectiveness of digital tools in research has yet to be thoroughly evaluated both in terms of individual tools (online surveys, audio message, social media, text-messaging) and the content delivered through these technologies. It is also important to assess the impact of digital tools in recruitment of underrepresented populations [4].

SMS text messaging has shown to be an efficient tool for improving response in population-based survey research. Text response rates were higher than telephone response rates [8]. Due to its ease of use and convenience, SMS facilitates participation in underrepresented groups [9]. Moreover, SMS allows for a representative response sample in a sensitive topic survey such as COVID-19 vaccine hesitation [10]. Not only does SMS facilitate survey research and sample quality, but it also allows the researcher to customize content for specific populations. For example, Plante et al. found that longer messages elicit a higher response rate than shorter ones during the recruitment phase in a community-based study [11]. Van den Berg et al. poses that inclusivity of the sample and thus participation of diverse groups can be improved by including descriptive social norms in the messages [4]. The use of social norms in message tailoring reflects key principles of message framing theory, which emphasizes how framing participation as a common and valued behavior can enhance engagement [12]. Thus, the attributes of SMS may make it a powerful digital tool for precision medicine.

In the present study, we assessed differences in likelihood of opening a recruitment message link and likelihood to consent to participate in a population-wide genomic screening study across four different recruitment messages delivered via text. We also assessed sociodemographic differences in open rate and consent rate by message type.

## Methods

### Overview of In Our DNA SC

*In Our DNA SC* is a large community-based research project launched in November 2021 by the Medical University of South Carolina (MUSC). The project’s goal is to collect population genomic data to better understand how DNA impacts health. Through a partnership with Helix, a California-based genomics company, those enrolled in the program received information about their genomic susceptibility to hereditary breast and ovarian cancer, hereditary colorectal cancer (Lynch Syndrome), familial hypercholesterolemia, and their genetic ancestry and traits. Anyone 18 years and older with the ability to read and write in English or Spanish with no history of allogenic bone marrow and/or stem cell transplants is eligible to join the program at no cost.

The recruitment process for this study included active and passive recruitment, including in-person events, electronic health records (EHR), patient portal messages (MyChart), SMS messaging and access to the study’s website. The initial recruitment strategy consisted of contacting patients whose MUSC medical records indicated no participation in *In Our DNA SC.* Potential participants received either an SMS text message or a message through MyChart that included a link to the project’s website [13] and instructions on how to enroll. To sign up, participants need to have an MUSC MyChart account or create one. Once logged into MyChart, participants can access and sign the consent form after answering some questions aimed at validating informed consent. In the current study, we evaluated the efficacy of a message based on the response of participants who received SMS text messages.

### Development of text message themes

Four distinct versions of recruitment messages were developed by the *In Our DNA SC* marketing team, then reviewed and endorsed by the project’s community advisory board (Table 1). Each message was designed to appeal to a different descriptive social norm about the importance of scientific research: generic, individual, communal, or research discoveries. We selected these message frames (individual impact, community impact, research discoveries) based on discussions with the community advisory board and participant feedback about what is most important to encourage participation. The generic version of the message had previously been developed and used to reach out to participants.

**Table 1.** Messaging types

Options	Message	Distinct Message Elements
Option 1: Generic	In Our DNA SC is a new community health research project at MUSC designed to look at how your DNA affects your health and improve access to personalized health care for our community. Once you participate, you and your doctor will receive confidential results about your genetic risk for certain cancers and heart disease at no cost to you. Visit the In Our DNA SC webpage (link) for details on how to enroll, participate and schedule your appointment for sample collection. Questions? Call ###-###-###.	N/A
Option 2: Individual Impact	MUSC’s In Our DNA SC community health research project is looking to provide confidential genetic testing for 100,000 South Carolinians at no cost to you. Once enrolled, you and your doctor will receive confidential results about your genetic risk for certain cancers and heart disease. This information could improve health care for you, your family, and our community. To get started, visit the In Our DNA SC webpage (link) for details on how to enroll, participate and schedule your appointment for sample collection. Questions? Call ###-###-###.	Emphasize “no cost to you” component of participation. State that the information “could improve health care for you, your family, and our community.”
Option 3: Community Impact	Want to support new research discoveries for our community while learning about how your DNA affects your health? Join In Our DNA SC, a new community health research project launched by MUSC! The project offers confidential results about your genetic risk for certain cancers and heart disease at no cost to you. You can visit the In Our DNA SC webpage (link) for details on how to enroll, participate and schedule your appointment for sample collection. Questions? Call ###-###-####.	State the opportunity to support “our community” via research discoveries
Option 4: Research Discoveries	Have you heard about MUSC’s community health research project, In Our DNA SC? The goal of this program is to improve access to personalized health care designed for you and to support new research discoveries in our community. The project offers confidential results about your genetic risk for certain cancers and heart disease at no cost to you. Visit the In Our DNA SC webpage (link) for details on how to enroll, participate and schedule your appointment for sample collection. Questions? Call ###-###-###.	State that participation will help “support new research discoveries”

### Population and recruitment approach

**Figure 1.**
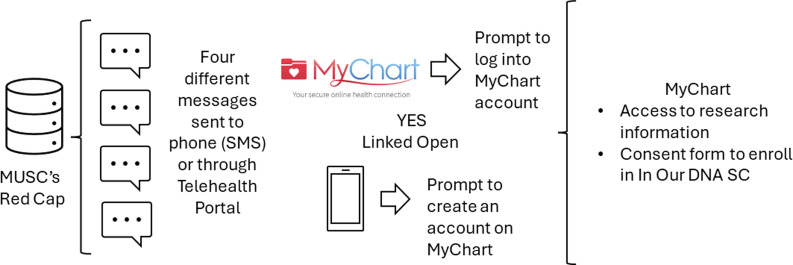
Population and recruitment approach.

15,977 individuals were randomly assigned to receive one of four messages: (1) generic, (2) individual impact, (3) community impact, (4) research discoveries (Figure 1). These individuals were identified through MUSC’s EHR and had not previously participated in *In Our DNA SC*. All messages included a link that directed them to MyChart where study information and the consent form was available. Two primary outcomes were measured: (1) open rate (date and time when individual opened the message), and (2) consent rate (after opening the message, whether the individual consented to In Our DNA SC). Sociodemographic factors of interest included sex, race, and age.

### Statistical analysis

We analyzed a sample of *n* = 15,977 potential participants who received a recruitment message through SMS from July 21,2023 until January 10, 2024. The 15,977 potential participants randomized to one of four messages, were selected from 146,228 potential participants using a stratified random sample based on gender, age category, and race-ethnicity group to ensure adequate sample size across the demographic characteristics of interest. The date and time when an individual opened one of the four messages and/or signed the consent form was recorded. The responses were then converted to binary, 1 if the message was opened and if participant enrolled, 0 otherwise. We included race, sex, and age as sociodemographic predictors. We also included the time lag between when a message was opened and enrollment as well as the number of attempts to recruit, i.e., the number of messages sent, to investigate which of the available factors aside from the message type had an impact on enrollment. Since our primary focus was to investigate how the language of each message affected participation, we also evaluated the cohort of participant who did not open the message to examine if a particular message had a significantly high negative effect. To assess the impact of the four different messages on recruitment we applied Pearson’s Chi-square Test to the proportion of messages both opened and those that resulted in consent. We also conducted descriptive statistics to evaluate demographic composition of different message groups based on response and participation. Finally, we explored the significance of demographics, time of response, and number of recruitment attempts using logistic regression models with different subsets of predictors. To interpret our results, we relied on the well-established elaboration likelihood model (ELM) from marketing research [14]. ELM posits that there are two ways a message can persuade the receiver: A *central route* where the receiver develops cognitive responses in reply to the ideas and concepts transmitted in the message, and a *peripheral route* in which the receiver’s perceptions about the product is formed from other sources not transmitted in the message [15].

## Results

Of the 15,977 individuals who received a text message, 2.4% opened the message (*n* = 382) and 0.8% enrolled (*n* = 135) (Table 2). Over one-third of individuals that opened the message enrolled in the study (*n* = 135, 35.3%). Females were more likely than males to open the initial message (3.1% vs. 1.6% open rate, *p* < 0.001) and consent (1.1% vs. 0.5% consent rate, *p* < 0.001). Individuals that identified as Hispanic were most likely to open the message than all other races (3.4%, *p* < 0.001) and consent to participate (1.6%, *p* < 0.001). Individuals between 30–39 were most likely to open the message (3.4%, *p* < 0.001) and individuals aged 40–49 were most likely to consent (1.6%, *p* < 0.001). The majority of participants consented to the study within 14 days (20,000 minutes) of receiving the message. On average, participants consented to the study within 1 day after opening the message. Over 75% of participants received one message, with some receiving up to 5. However, the number of attempts to recruit had no significance according to the logistic model.

**Table 2.** Sociodemographic characteristics of sample

	Total Population	Opened Message		Consented	
	*n* = 15,977	*n* = 382	%	*n* = 135	%
Female	8192	254	3.1%	94	1.1%
Male	7785	128	1.6%	41	0.5%
Black	*3468*	*41*	1.2%	11	0.3%
White	*3476*	*108*	3.1%	39	1.1%
Hispanic	*2395*	*81*	3.4%	39	1.6%
Other	*3184*	*87*	2.7%	24	0.7%
Unknown	*3454*	*65*	1.9%	22	0.6%
Age 18–29	2887	52	1.8%	17	0.6%
Age 30–39	2785	95	3.4%	38	1.4%
Age 40–49	2740	91	3.3%	43	1.6%
Age 50–59	2665	60	2.3%	14	0.5%
Age 60–69	2533	49	1.9%	13	0.5%
≥70	2367	35	1.5%	10	0.4%

We next assessed whether the type of message was significantly associated with open rate or consent rate (Table 3). We did not find significant differences in open rate (*p* = 0.2521) or consent rate (*p* = 0.5392) across the four message types.

**Table 3.** Open rate and consent rate by message type

Message	Opened Message (*n* = 382)	% opened	Consented of opened (*n* = 135)	% of those that opened consented	Consented of Total Sent (*n* = 135)
1 (*n* = 3982)	92	2.3%	28	30.4%	0.7%
2 (*n* = 3990)	110	2.8%	40	36.4%	1%
3 (*n* = 3994)	83	2.1%	34	41.0%	0.9%
4 (*n* = 4011)	97	2.4%	33	34.0%	0.8%

We then assessed differences in likelihood to open messages and consent by sociodemographic factors (Table 4). Females were most likely to open message 2 (*n* = 78, 3.9%) than all other message types (*p* = 0.04837). Once opened, females were most likely to consent to message 2 (*n* = 31, 39.7%) than all other message types (*p* = 0.001416). There were no significant differences in message type opened by race or by age.

**Table 4.** Sociodemographic differences in open rate and consent rate

	OPENED MESSAGES (*n* = 382)								CONSENTED of those that Opened (*n* = 135)							
	Message Option								Message Option							
	1 (*n* = 92)		2 (*n* = 110)		3 (*n* = 83)		4 (*n* = 97)		1 (*n* = 28)		2 (*n* = 40)		3 (*n* = 34)		4 (*n* = 33)	
		%	*n*	%	*n*	%	*n*	%	*n*	%	*n*	%	*n*	%	*n*	%
Female	60	3.0	78	3.9	48	2.4	68	3.4	17	28.3	31	39.7	21	43.8	25	36.8
Male	32	1.7	32	1.7	35	1.8	29	1.5	11	34.4	9	28.1	13	37.1	8	27.6
Black	12	1.4	10	1.2	12	1.4	7	0.8	3	25.0	3	30.0	2	16.7	3	42.9
White	26	3.1	26	3.4	25	2.1	31	2.8	11	42.3	8	30.8	12	48.0	8	25.8
Hispanic	14	2.4	23	6.1	21	3.6	23	3.9	7	50.0	15	65.2	9	42.9	8	34.8
Other	23	3.0	26	3.4	16	2.1	22	2.8	2	8.7	9	34.6	5	31.3	8	36.4
Unknown	17	2.0	25	2.9	9	1.1	14	1.6	5	29.4	5	20.0	6	66.7	6	42.9
Age 18–29	9	1.3	19	2.7	12	1.7	12	1.7	1	11.1	9	47.4	3	25.0	4	33.3
Age 30–39	17	2.5	30	4.5	20	2.9	28	4.2	6	35.3	13	43.3	9	45.0	10	35.7
Age 40–49	33	5.1	21	3.2	16	2.4	21	3.1	13	39.4	10	47.6	11	68.8	9	42.9
Age 50–59	15	2.3	17	2.6	17	2.6	11	1.7	4	26.7	4	23.5	4	23.5	2	18.2
Age 60–69	7	1.1	17	2.8	10	1.6	15	2.4	2	28.6	3	17.7	3	30.0	5	33.3
≥70	11	1.9	6	1.0	8	1.4	10	1.7	2	18.2	1	16.7	4	50.0	3	30.0

Finally, we investigated the sample of unopened messages by message type. There were no statistically significant differences between unopened messages by sex, age category, nor race based on the Pearson Chi-Square Test.

The logistic regression model indicated that the likelihood for opening a message was statistically significant for females (males’ coefficient was -0.63 with a *p*-value < 0.0010), A female that opened a message was likely to be Hispanic (*p* < 0.01) and between 30 and 39 years old (*p* = 0.027).

## Discussion

### Differences in open rate and consent rate across tailored messages

We found no significant differences in open rate or consent rate based on message type across our full sample of participants. In a similar message condition study, researchers assessed differences between a general message and tailored message on likelihood to consent and return their saliva sample for a genetic study. No differences were found in participation between those that received a general message compared to a tailored message [16].

When assessing differences in responsiveness to messages across message type, the only differences we found in message type was by gender, with no differences in message open rate or consent rate across race or age. We found that females were more likely than males to open the initial message (3.1% vs. 1.6%) and consent (1.1% vs. 0.5%). Females were also most likely to open message 2 (3.9%) compared to all other message types and most likely to consent to message 2 once opened (39.7%) compared to all other message types. Message option 2 was focused on “individual impact” which included standard information about *In Our DNA SC*, as well as details about the impact of receiving results, which was framed as, “you and your doctor will receive confidential results about your genetic risk for certain cancers and heart disease.” The message also indicated that the results could “improve health care for you, your family, and our community.” Prior literature has identified perceived value of the information – both clinical and personal utility – as key motivators for participation in genome sequencing. Message 2, which was most salient among females focuses on demonstrating both the clinical utility (ability to impact healthcare) and personal utility (motivation based on information that increases perceived empowerment and understanding of personally relevant information). In particular, personal utility has been identified as a motivator for participation in genomics research, regardless of clinical utility or actionability [21,22].

### Effectiveness of SMS in recruitment to PGS

We found overall low open rates and consent of individuals contacted via SMS text in our population-based genomic screening study. The overall open rate was 2.4% and a total of 0.8% of those that received a text message consented. Still, the response rate was higher than the 1.7% found in a similar recruitment study [4].

The effectiveness of using SMS in recruitment for our PGS program reveals both promises and challenges. Digital approaches, such as SMS, have been suggested to help balance the need to recruit large groups of individuals with the need to develop personalized, tailored messages. This challenge is particularly salient in recruitment for genetic studies where recruitment of large, representative samples is necessary to detect genetic signals of small effect [16]. In another study, 1,000 cold text survey invitations were sent and a total of 7% began the survey and 3.6% completed it. Notably, 17.2% of individuals who were contacted for this study unsubscribed. Similar to our findings, respondents most commonly came on the same day that the initial message was sent (88%). The most common reason for non-response in the prior study included: not understanding the source or reason for text (30%) and being busy or occupied (27%) [17].

Other considerations for low response rate include frequency of messages and the modality of message delivery. Other recruitment initiatives have demonstrated significant differences in likelihood to participate in colorectal cancer screening between one-time text message reminders (2.3%) and sequenced communication (19.6%) [18]. We did not attempt multiple outreach attempts via text message due to the large pool of participants. Aside from frequency of outreach, opportunities exist to assess combinations of approaches (e.g., single digital approach vs. combined digital approach and non-digital approach vs. multiple combined digital approaches) [19,20].

Regarding effectiveness of tailored messages, we based our interpretation on the ELM model. In this framework, the messages options are the *central route,* through which we encoded different values related to research: a neutral or generic message (Option 1), individual benefits (Option 2), scientific impact in the community (Option 3**)**, and the value of research (Option 4). Our results indicated that the topics included in the tested messages did not elicit a response, but a participant’s age, race and gender were more relevant for participants to open the link.

### Limitations

Limitations included a low response rate across our population. This may have been due to message delivery method, timing, or participant fatigue. For instance, our program sent a one-time text message, and we did not assess reasons for non-response. In addition, the generalization of our findings may be limited, given the specific population of patients in one health system and focus on recruitment for a large-scale population-wide genomic screening program.

In interpreting the impact of the four different messages, we did not conduct cognitive interviews to evaluate whether the target audience perceived them as different. Participant feedback on the messages was collected informally through community advisory board meetings.

## Conclusions

Our findings indicate that the current variation in the SMS text messages had no impact on recruitment and open or consent rate for *In Our DNA SC*, a large population-based genomic screening project. Our results highlight an overall low response rate and limited variation in effectiveness across SMS message content, emphasizing the opportunity for more inclusive and innovative recruitment strategies. There is a continued need to explore more personalized approaches for recruitment into research studies. Low response rate can be addressed by including a combination of different delivery methods: SMS. Web, e-mail as well as sending reminders to potential participants. Future research could also explore more personalized and interactive messages. To do so, it may be necessary to survey target groups about their perception and sentiment regarding a particular study and to test, a priori, the efficacy and perception of different messages’ content on the target audience. Moreover, implementing differentiated SMS messaging within a tiered, sequential deployment approach (email, SMS, portal, other), along with repeated invitations may increase the likelihood of message opening and response.

Ensuring equity in research recruitment remains critical, with a focus on tailoring digital recruitment tools to underrepresented communities, while ensuring scalability of approaches for population-level initiatives, such as population-based genomic screening.
